# Chalcogen ‘like-like’ Interactions Involving Trisulphide and Triselenide Compounds: A Combined CSD and Ab Initio Study

**DOI:** 10.3390/molecules23030699

**Published:** 2018-03-19

**Authors:** Antonio Bauzá, Antonio Frontera

**Affiliations:** Department of Chemistry, Universitat de les Illes Balears, Crta de Valldemossa km 7.5, 07122 Palma de Mallorca (Baleares), Spain

**Keywords:** chalcogen bonding interactions, CSD search, RI-MP2 calculations, AIM and NBO analyses

## Abstract

In this manuscript, we combined a search in the Cambridge Structural Database (CSD) and ab initio calculations (RI-MP2/def2-TZVPD level of theory) to analyze the ability of trisulphide and triselenide moieties to establish chalcogen ‘like-like’ interactions. A preliminary CSD inspection revealed two predominant structural patterns, depending on the *anti* or *syn* conformation adopted by the substituents of the S_3_/Se_3_ bridge, leading to bifurcated or double chalcogen bonding interactions, respectively. In order to analyze these two relevant structural motifs we have used a series of S and Se derivatives Ch_3_X_2_ (Ch = S and Se and X = H, F, CN, and CF_3_) which act as both electron donor (using the lone pairs) and acceptor (using the σ-holes) entities. Besides, we have carried out “atoms in molecules” (AIM) and natural bonding orbital (NBO) analyses to further describe and characterize the chalcogen bonding interactions described herein. As far as we know, chalcogen···chalcogen interactions involving trichalconides (S_3_/Se_3_) have not been previously described in literature a may be of great importance in the preparation and characterization of new solids based on this subclass of σ-hole bonding.

## 1. Introduction

The conjunction of a great deal of noncovalent forces is considered of great importance for the advance and progress in the field of Supramolecular chemistry [1,2]. A depth comprehension of them is vital for chemists working in this discipline, since many chemical and biological processes are governed by an intricate combination of noncovalent interactions, settling the basis of highly specific recognition processes. For instance, interactions between hosts and guests cover the formation of novel supramolecular assemblies presenting high affinities even in highly competitive media [3,4,5,6]. For this reason, a proper description and understanding of noncovalent interactions between molecules is key for success in this field of research. One of the classical and well-known supramolecular forces present in many chemical and biological environments is hydrogen bonding [7]. In a parallel way, halogen bonding [8] is a noncovalent force that shares strength and directionality features with the hydrogen bonding interaction. Consequently, a series of studies using the Cambridge Structural Database (CSD) were carried out to shed light into the impact of this interaction in solid state chemistry [9]. It is also widely recognized that σ-holes can also appear in positive electrostatic potential regions of covalently bond atoms of groups III to VIII [10,11,12,13]. In addition, several theoretical studies have been devoted to study their physical nature [14,15,16,17,18], concluding that it is basically explained by the interaction of an electron rich entity (electron donor) with a σ-hole (electron acceptor), in a parallel way to hydrogen and halogen bonding interactions [19,20]. More in particular, chalcogen bonding interactions involving elements from group VI have been widely analyzed in a series of both experimental and theoretical studies [21,22,23,24,25,26,27]. Interestingly, diallyl trisulphide (DATS) [28], also known as Allitridin, is an organosulfur compound responsible for many health benefits of garlic. These include anti-cancer effects, since DATS has been shown to be involved in the apoptosis of cancer cells and a decrease in cancer cell proliferation [29,30], platelet aggregation, blood pressure reduction, decreases in cholesterol levels, and increases in levels of reactive oxygen species (ROS) [31,32]. Finally, DATS is considered a promising tool in the treatment of cardiac arrhythmias through its ability to regulate the human hERG (ether-à-go-go-related gene) channel, which is a pore-forming subunit of potassium channels responsible for the creation of delayed rectifier potassium ion currents in many cells, including cardiac myocytes [33].

**Figure 1:**
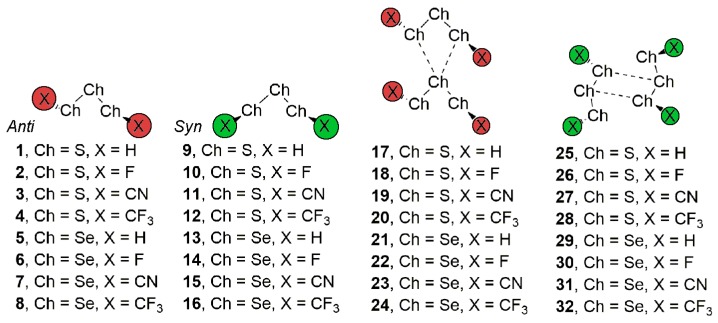
Compounds **1**–**16** and complexes **17** to **32** studied in this work.

In this regard, owing to the potential dual behavior of trisulphides (and also triselenides) as both chalcogen bond donor and acceptor moieties, we performed an initial inspection in the CSD [34] and found many X-ray structures where chalcogen ‘like-like’ interactions dominated their supramolecular architecture. More in detail, we identified two predominant structural patterns, which depend on the *anti* or *syn* conformation adopted by the substituents of the S_3_/Se_3_ moiety (see Figure 1). Consequently, we designed a theoretical study in order to analyze the energetic and stability properties of these two structural motifs. For this purpose, we used Ch_3_X_2_ (Ch = S and Se and X = H, F, CN, and CF_3_) moieties as both electron and σ-hole donors. In addition, we have performed “atoms in molecules” (AIM) and natural bonding orbital (NBO) analyses to further characterize the interactions described herein. As far as our knowledge extends, chalcogen bonding interactions involving trisulphide and triselenide moieties have not been previously reported in literature and may represent and interesting topic for those chemists working in the field of chalcogen chemistry, more in particular, in the preparation of organosulfur and organoselenide derivatives.

## 2. Results and Discussion

### 2.1. Cambridge Structural Database Search

**Figure 2:**
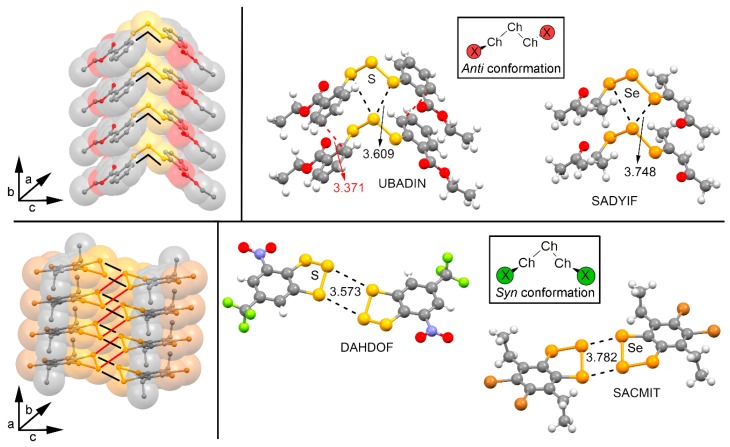
(**Left**) Structural patterns observed for *anti* (**top**) and *syn* (**bottom**) substituted S_3_/Se_3_ compounds. (**Right**) Partial views of the X-ray structure of some *anti* (**top**) and *syn* (**bottom**) trisulphide and triselenide compounds exhibiting chalcogen ‘like-like’ interactions. The Cambridge Structural Database (CSD) codes are indicated. Distances in Å.

We have firstly explored the CSD (version 5.38, updated February 2017) to find evidence of the ability of trisulphide and triselenide compounds to establish chalcogen ‘like-like’ interactions. For the search, we have retrieved all trisulphide and triselenide compounds from CSD with the unique restriction that the three chalcogen atoms in the molecule are divalent (bonded to two atoms). We have found 123 trisulphide compounds and 36 triselenide compounds. Among these, in 10 trisulphide and 8 triselenide structures the crystal packing is governed by chalcogen bonding interactions that follow the two recognition patterns shown in Figure 2. First, in case of the *anti* conformation (UBADIN [35] and SADYIF [36] structures), the crystal packing is formed by 1D infinite columns disposed in an ‘arrow-like’ fashion, which is stabilized by the formation of bifurcated chalcogen ‘like-like’ interactions involving a central S/Se atom of one molecule acting as chalcogen bond donor and the lone pairs of the two vicinal S/Se atoms present in the other unit as electron donor moieties. In addition, in UBADIN the aromatic substituents interact by means of ancillary π–π stacking interactions (highlighted in red in Figure 2). On the other hand, in SACMIT [37] and DAHDOF [38] structures, the substituents of the S_3_/Se_3_ moiety are oriented in *syn* conformation, leading to the establishment of double chalcogen bonds, thus conferring a completely different solid state architecture dominated by the formation of ‘zig-zag’ self-assembled dimers. More in detail, each moiety acts as both electron donor and acceptor entity by using the lone pairs of the central S/Se atom and one of the σ-holes present in a vicinal S/Se atom. In order to analyze the energetic and geometrical parameters of both structural patters we have performed a theoretical study using the compounds shown in Figure 1 (see above).

### 2.2. Preliminary MEP Analysis

**Figure 3:**
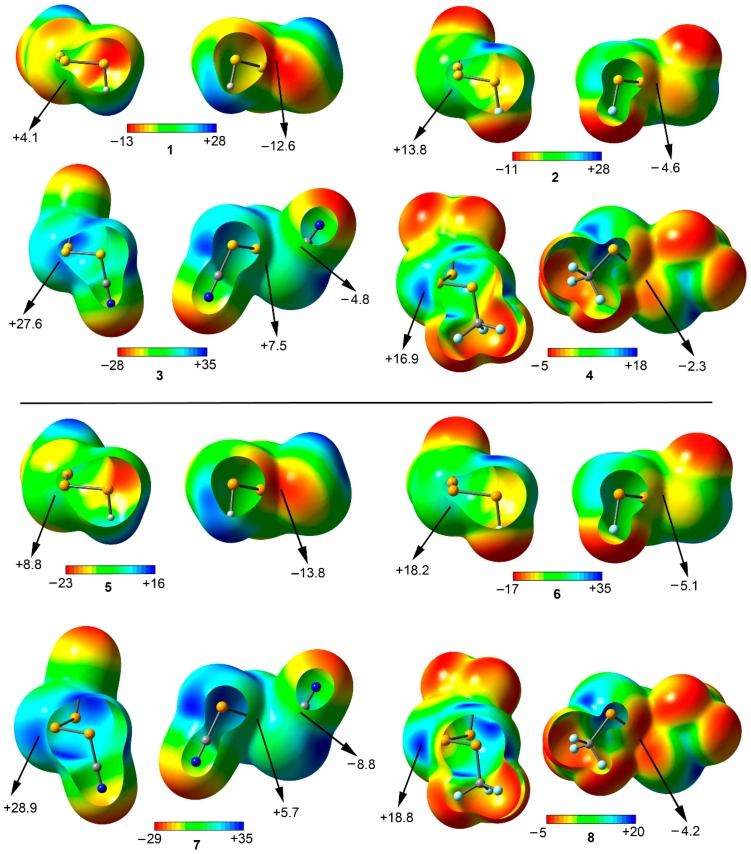
Molecular electrostatic potential (MEP) surfaces of compounds **1** to **8** used in the study. Energies at selected points of the surface (0.001 atomic units (a.u.)) are given in kcal/mol.

**Figure 4:**
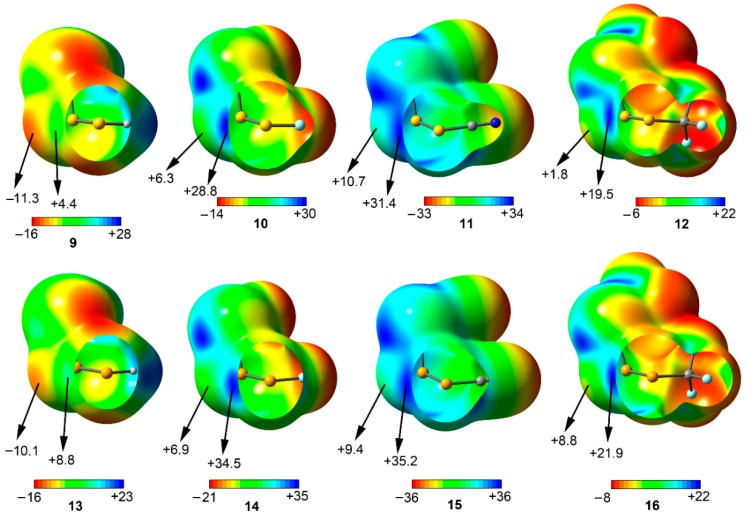
MEP surfaces of compounds **9** to **16** used in the study. Energies at selected points of the surface (0.001 a.u.) are given in kcal/mol.

We have firstly computed the molecular electrostatic potential (MEP) mapped onto the van der Waals surface for compounds **1** to **16** (Figure 3 and Figure 4). Among the *anti* compounds **1** to **8** two positive electrostatic potential regions are found on the extension of both the X−Se (X = H, F, CN, and CF_3_) and S−S bonds, named σ-holes. The presence of these regions ensures an attractive interaction with electron rich entities from an electrostatic point of view. In addition, the MEP values become more positive as the electron-acceptor ability of the substituent does (H < F < CF_3_ < CN), as it is commonly known for other σ-hole interactions [10]. Furthermore, the MEP values are more positive for compounds involving Se (**5** to **8**) than for those involving S (**1** to **4**), thus initially expecting larger interaction energy values for complexes involving the former from an electrostatic perspective. Moreover, for compounds **1** to **3** and **5** to **7** a negative electrostatic potential region appears at the rear part of the molecule due to the presence of the lone pairs belonging to the two vicinal S/Se atoms, making these molecules suitable for acting also as electron donor entities. It is also worthy to mention that in case of compounds **4** and **8**, the MEP value over this region is positive, however it becomes negative over the π-cloud of the CN group. From the inspection of these results, it is expected that complexes involving **3** and **7** present the most favorable interaction energy values from an electrostatic perspective since they showed the most positive σ-hole MEP values as well as moderately negative MEP value over the CN π-system. Moreover, for complexes involving F and CF_3_ substituted compounds (**2**, **4**, **6**, and **8**) a similar strength upon complexation is expected owing to their respective σ-hole and lone pair MEP values. Finally, compounds **1** and **5** present on the one hand the most negative lone pair MEP values and on the other hand the less positive σ-hole MEP values.

Among the *syn* compounds (**9** to **16**) the σ-hole MEP values of the two vicinal S/Se atoms are positive in all cases following the behavior H < CF_3_ < F < CN, similar to that observed for compounds **1** to **8**. In addition, the σ-hole MEP values for the Se derivatives (**13** to **16**) are more positive than the ones obtained for S compounds **9** to **12**, in agreement to that obtained for the *anti* set. In case of the central S/Se atom, the MEP value is negative in case of compounds **9** and **13**, however, it becomes positive when attaching a strong electron-acceptor substituent (compounds **10** to **12** and **14** to **16**), thus expecting weaker binding energy values from an electrostatic perspective (especially in complexes involving compounds **11**, **15**, and **16**). From the inspection of the results, complexes involving **9** and **12** are expected to present stronger interaction energy values than their F, CN, and CF_3_ analogous, owing to their respective σ-hole and lone pair MEP values. Finally, for the rest of the compounds (**10** to **12** and **14** to **16**) other energy components, such as dispersion and induction terms are meant to play a key role in the stabilization of their respective complexes.

### 2.3. Energetic and Geometric Results

**Figure 5:**
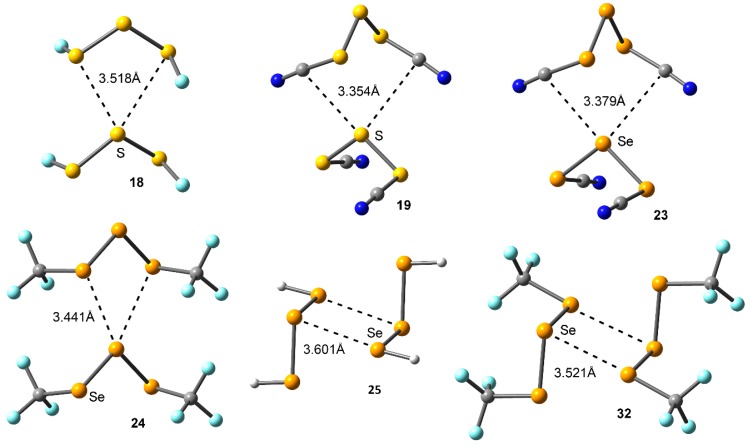
Optimized geometries of some representative complexes at the RI-MP2/def2-TZVPD level of theory.

Table 1 gathers the interaction energies and equilibrium distances of the optimized complexes **17** to **32** (see Figure 1 and Figure 5) computed at the RI-MP2/def2-TZVPD level of theory. From the analysis of the results, several points are worthy to discuss. First, in all cases negative and moderately strong interaction energy values were obtained, ranging from −7.1 to −2.1 kcal/mol. Second, *anti* complexes involving Se (**21**–**24**) obtained larger interaction energy values than those involving S (**17**–**20**), as expected from the MEP analysis discussed above. In case of *syn* complexes, the same energetic behavior is observed, obtaining larger binding energy values for Se complexes **29** to **32** compared to their S analogous (**25** to **28**). Third, *syn* complexes (**25** to **32**) achieved larger interaction energy values than those presenting the *anti* conformation (**17** to **24**), since double chalcogen bonding interactions instead of bifurcated ones are established in the former.

For complexes involving *anti* conformation (**17** to **24**) the two σ-holes of the central S/Se atom are pointing towards the lone pairs of the vicinal S/Se atoms present in the other unit, thus establishing bifurcated chalcogen bonding interactions, in agreement with the X-ray structures shown above. However, in complexes **19** and **23** the σ-holes of the S/Se atom are pointing to the π−system of the CN group, which showed a negative MEP value (see Figure 4). Precisely, these complexes achieved the largest interaction energy values of their respective sets (–4.6 and –5.5 kcal/mol, respectively), followed by complexes **20** and **24** where the CF_3_ group acts as substituent (−3.7 and −4.9 kcal/mol, respectively). Finally, complexes **17**, **18**, **21**, and **22** involving H and F obtained slightly similar interaction energy values (i.e., −2.1 and −2.3 kcal/mol for complexes **17** and **18**, respectively), contrary to the MEP analysis discussed above, likely due to a compensating effect between the σ-hole and lone pair MEP values.

Among the *syn* complexes considered (**25** to **32**) a different behavior is observed depending on the chalcogen atom used. For S complexes (**25** to **28**), complex **28** involving CF_3_ group achieved the most favorable interaction energy value (−4.4 kcal/mol). This result is somewhat unexpected, since compound **4** involving CF_3_ does not present the most positive σ-hole values, however, it may be compensated with a less positive lone pair MEP value than compounds **10** and **11** involving F and CN groups. In addition, among complexes **25** and **26** involving H and F, almost identical binding energy values were obtained (−3.7 and −3.6 kcal/mol, respectively), which may also be due to a compensating effect between the more positive σ-hole MEP value observed in compound **10** and the more negative lone pair MEP value obtained for **9**. Finally, complex **27** involving CN obtained the poorest interaction energy value (−3.0 kcal/mol), since it presents the most positive lone pair and σ-hole MEP values of the series. For Se complexes (**29** to **32**) complex **30** involving F obtained the largest interaction energy value of the study (−7.1 kcal/mol). This issue will be further described in the NBO analysis (see Table 2). In addition, complexes **29** and **32** involving H and CF_3_ groups obtained similar interaction energy values (−5.0 and −5.8, respectively). Finally, complex **31** involving CN obtained the poorest interaction energy value of the set (−4.3 kcal/mol), similarly to S series (complex **27**).

### 2.4. “Atom in Molecules” and Natural Bond Order Analyses

**Figure 6:**
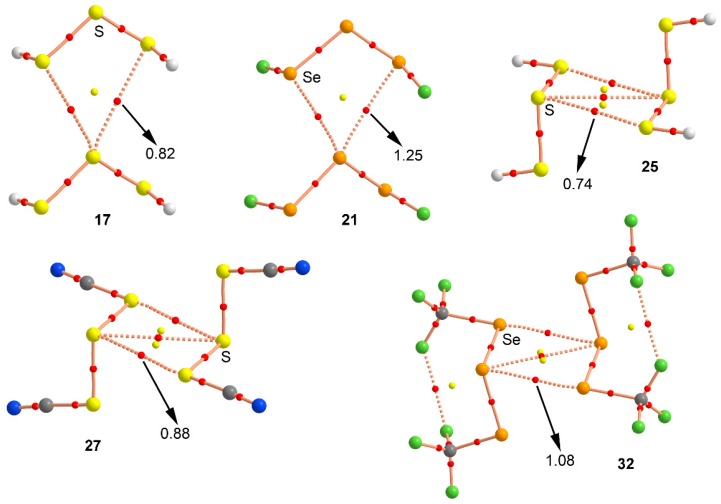
Distribution of critical points (red spheres) and bond paths for complexes **17**, **21**, **25**, **27**, and **32** at the MP2/def2-TZVP level of theory. Bond and ring CPs are represented by red and yellow spheres, respectively. The values of the charge density (ρ) at the bond critical points that emerge upon complexation are indicated in a.u.

We have used the Bader’s theory of “atoms in molecules” [39] (AIM) to characterize the noncovalent interactions present in complexes **17**–**32**. A bond critical point (CP) and a bond path connecting two atoms is an explicit indication of interaction. The AIM distribution of critical points and bond paths computed for all complexes is shown in Figure 6. As noted, in case of the *anti* complexes **17** and **21** the bifurcated chalcogen bonding interaction is characterized by the presence of two symmetrically distributed bond CP and a bond paths connecting the σ-holes of the central S/Se atom with the lone pairs of the two vicinal chalcogen atoms present in the other molecule. The interaction is further described by the presence of a ring CP as a consequence of the formation of a supramolecular ring. On the other hand, for *syn* complexes **25**, **27**, and **32** the double chalcogen bonding interactions are characterized by the presence of two bond CPs and bond paths connecting the central and vicinal S/Se atoms of both moieties. In addition, two ring CPs are formed due to the presence of two supramolecular rings. It is also worth mentioning that, in complex **32**, two bond CPs connect the fluorine atoms of the two CF_3_ groups present in each moiety, thus characterizing intramolecular F···F contacts. Finally, the value of the Laplacian at the bond critical points in all cases is positive, as it is commonly found in closed shell interactions.

In order to study if orbital contributions are important to explain the chalcogen bond complexes described above, we have performed NBO calculations focusing our attention on the second order perturbation analysis, due to its usefulness in the analysis of donor–acceptor interactions [40]. The results for some representative complexes are summarized in Table 2 and some points are worthy to remark. First, for *anti* complexes **17** to **24** the main orbital contribution comes from the interaction between the lone pairs (LP) of the S/Se atoms and an antibonding (BD*) S–S orbital with the exception of complexes **19** and **23** where it comes from the donation of bonding (BD) C–N orbital to an BD* S–S orbital. In addition, for these complexes an additional orbital contribution from the lone pairs (LP) of the S/Se atom to an BD* S–S orbital is also observed. On the other hand, in case of *syn* complexes **25** to **32**, the main orbital contribution is attributed to the interaction between the lone pairs (LP) of the S/Se atoms and an BD* S/Se–X (X = H, F and C) orbital. Particularly, complex **30** involving F presents the largest orbital contribution of the study, in agreement with the binding energy value obtained (see above). It can also be noted that the orbital contributions for the Se complexes (**21** to **24** and **29** to **32**) are larger in magnitude than for S involving complexes (**17** to **20** and **25** to **28**), in line with the interaction energy values discussed above. However, a clear behavior cannot be established when comparing the magnitude of the orbital contributions between *anti* and *syn* complexes. Finally, it is also worth pointing out that the magnitude of the orbital contributions ranges from moderate (~30% for complex **20** and **26**, ~20% for complexes **19** and **29**) to strong (~50% for complex **17** and ~80% for complexes **30** and **31**) compared to the total interaction energy, remarking the importance of orbital interactions in the global stabilization of the complexes studied herein.

## 3. Theoretical Methods

The geometries of the complexes studied herein have been fully optimized at the RI-MP2/def2-TZVPD level of theory. The RI-MP2 has been validated as an efficient and reliable method for the treatment of chalcogen bonding interactions among others [41]. The calculations have been performed by using the program TURBOMOLE version 7.0 (TURBOMOLE GmbH, Karlsruhe, Germany) [42]. The C_2_ and C_i_ symmetry point group have been used in the optimization of the complexes. The interaction energies were calculated with correction for the basis set superposition error (BSSE) by using the Boys–Bernardi counterpoise technique [43]. Frequency calculations were performed at the RI-MP2/def2-TZVPD level of theory. The NBO analysis has been carried out at the HF/def2-TZVP level of theory. Bader’s “atoms in molecules” theory has been used to study the interactions discussed herein by means of the AIMAll calculation package [44]. The calculations for the wavefunction and NBO analyses have been performed by means of the Gaussian 09 calculation package [45].

## 4. Conclusions

In this manuscript, we have theoretically analyzed (RI-MP2/def2-TZVPD) the chalcogen ‘like-like’ interactions established between trisulphide and triselenide compounds. A preliminary inspection of the CSD database revealed two predominant structural motifs, where the substituents of the S_3_/Se_3_ bridge were disposed in either *anti* or *syn* conformation. Consequently, we designed a computational study in order to analyze the energetic and geometrical parameters of these two relevant crystal patterns. More in detail, we have used Ch_3_X_2_ (Ch = S and Se, X = H, F, CN, and CF_3_) derivatives, which act as both σ-hole and lone pair donors. In addition, we have also performed AIM and NBO analyses to further characterize the interactions described above. In this regard, orbital interactions involving the lone pairs and σ-holes of both S/Se moieties range from a moderate to a strong source of stability of the complexes studied. To the best of our knowledge, chalcogen bonding interactions involving S_3_/Se_3_ compounds have not been previously reported and may be important to understand the crystal packing phenomena of this family of compounds as well as in the preparation of novel chalcogenide derivatives.

## Figures and Tables

**Table 1:** Interaction energies with basis set superposition error (BSSE) correction (ΔE_BSSE_ in kcal/mol), equilibrium distances (R, Å), value of the density at the bond critical point (CP) (10^2^ x ρ, a.u.) and number of imaginary frequencies (N_imag_) for complexes **17**–**32** at the RI-MP2/def2-TZVPD level of theory.

Complex	ΔE_BSSE_	R	10^2^ x ρ	N_imag_
**17**	−2.1	3.546	0.82	0
**18**	−2.3	3.518	0.84	0
**19**	−4.6	3.577	0.73	0
**20**	−3.7	3.354	0.77	0
**21**	−3.7	3.419	1.38	0
**22**	−3.0	3.479	1.25	0
**23**	−5.5	3.441	0.83	0
**24**	−4.9	3.342	1.34	0
**25**	−3.7	3.546	0.74	0
**26**	−3.6	3.278	1.17	1
**27**	−3.0	3.482	0.88	1
**28**	−4.4	3.452	0.83	0
**29**	−5.0	3.601	0.93	1
**30**	−7.1	2.996	2.76	0
**31**	−4.3	3.521	1.20	1
**32**	−5.8	3.457	1.08	1

**Table 2:** Donor and acceptor natural bond orbital (NBO) interactions with indication of the second-order interaction energy E^(2)^ (kcal/mol) for complexes **17**–**32**.

Complex	Donor ^a^	Acceptor	E^(2)^
**17**	LP S	BD* S–S	1.12
**18**	LP S	BD* S–S	1.08
**19**	BD C–N	BD* S–S	0.84
	LP Se	BD* S–S	0.22
**20**	LP S	BD* S–S	0.94
**21**	LP Se	BD* Se–Se	3.62
**22**	LP Se	BD* Se–Se	2.70
**23**	BD C–N	BD* Se–Se	1.44
	LP Se	BD* Se–Se	0.60
**24**	LP Se	BD* Se–Se	3.46
**25**	LP S	BD* S–H	0.36
**26**	LP S	BD* S–F	1.08
**27**	LP S	BD* S–C	0.54
**28**	LP S	BD* S–C	0.36
**29**	LP Se	BD* Se–H	0.78
**30**	LP Se	BD* Se–F	5.40
**31**	LP Se	BD* Se–C	1.44
**32**	LP Se	BD* Se–C	1.28

^a^ LP, BD, and BD* stand for lone pair, bonding, and anti-bonding orbital, respectively.

## Supplementary Materials

The following are available online, cartesian coordinates of the complexes and results from the CSD search.
