# Supplementary material for: Chalcogen ‘like-like’ Interactions Involving Trisulphide and Triselenide Compounds: A Combined CSD and Ab Initio Study
Source: Molecules. 2018 Mar 19;23(3):699. doi: 10.3390/molecules23030699 (PMC6017106; doi:10.3390/molecules23030699)
Supplement: Supplementary file 1 [file molecules-23-00699-s001.pdf]

Chalcogen "like-like" interactions involving trisulphide and triselenide compounds: A combined CSD and *ab initio* study.

Antonio Bauzá,[1*] and Antonio Frontera,[1*]

[1]Department of Chemistry Universitat de les Illes Balears, Crta. de Valldemossa km 7.5, 07122 Palma (Baleares), Spain. E-mail: antonio.bauza@uib.es, toni.frontera@uib.es;

Fax: +34 971 173426

Electronic Supplementary Information

Cartesian Coordinates

17.

S	0.1609557	-1.6450298	-2.3460891
S	0.0000000	0.0000000	-1.1207018
S	-0.1609557	1.6450298	-2.3460891
S	0.0867532	-1.6576089	2.0129659
S	-0.0000000	0.0000000	3.2232328
S	-0.0867532	1.6576089	2.0129659
H	-1.2325961	-1.8219098	1.8218696
H	1.2325961	1.8219098	1.8218696
H	-1.1500608	-1.8631774	-2.5400120
H	1.1500608	1.8631774	-2.5400120

18.

S	0.7828724	-1.4279356	-2.2443663
S	0.0000000	0.0000000	-1.0649404
S	-0.7828724	1.4279356	-2.2443663
S	0.4845964	-1.5604146	2.0510415
S	0.0000000	0.0000000	3.2228009

S	-0.4845964	1.5604146	2.0510415
F	0.9553437	2.2101657	1.6649279
F	0.4897391	2.3957546	-2.5505333
F	-0.4897391	-2.3957546	-2.5505333
F	-0.9553437	-2.2101657	1.6649279

19.

S	0.0039999	-1.6456319	-2.0095773
S	0.0000000	0.0000000	-0.7733544
S	-0.0039999	1.6456319	-2.0095773
S	1.6164747	0.2157213	2.4425530
S	0.0000000	-0.0000000	3.7038040
S	-1.6164747	-0.2157213	2.4425530
C	-1.6508483	-1.7552055	-2.3461409
C	1.6962442	-1.3408207	1.7901392
C	-1.6962442	1.3408207	1.7901392
C	1.6508483	1.7552055	-2.3461409
N	-2.7957016	-1.8522145	-2.6043090
N	1.7194601	-2.3941902	1.2621103
N	-1.7194601	2.3941902	1.2621103
N	2.7957016	1.8522145	-2.6043090

20.

S	0.9845044	-1.3057042	-2.1009938
S	0.0000000	-0.0000000	-0.8743143
S	-0.9845044	1.3057042	-2.1009938
S	1.1776331	-1.1346960	2.3070784
S	0.0000000	-0.0000000	3.5321798
S	-1.1776331	1.1346960	2.3070784

C	0.3372519	2.5252729	-2.3811516
C	-0.3372519	-2.5252729	-2.3811516
C	0.0967155	-2.5878479	2.1155815
C	-0.0967155	2.5878479	2.1155815
F	-0.7727354	-3.0887366	-1.2559913
F	-1.3953681	-1.9945894	-2.9981902
F	0.1736295	-3.4806475	-3.1640349
F	0.7336378	-3.4412945	1.3097647
F	-1.0788432	-2.2781945	1.5670635
F	-0.1505461	-3.2024149	3.2719410
F	1.0788432	2.2781945	1.5670635
F	-0.7336378	3.4412945	1.3097647
F	0.1505461	3.2024149	3.2719410
F	0.7727354	3.0887366	-1.2559913
F	1.3953681	1.9945894	-2.9981902
F	-0.1736295	3.4806475	-3.1640349

21.

H	-1.3447800	-2.0159188	1.7621213
H	1.3447800	2.0159188	1.7621213
H	-1.2713323	-2.0366211	-2.5210115
H	1.2713323	2.0366211	-2.5210115
Se	0.0000000	-0.0000000	-0.9372575
Se	-0.1703989	1.8365674	-2.3568824
Se	0.1703989	-1.8365674	-2.3568824
Se	0.1025376	-1.8597442	1.9292861
Se	0.0000000	0.0000000	3.3102305
Se	-0.1025376	1.8597442	1.9292861

22.

Se	0.8950772	-1.5790344	-2.3190480
Se	0.0000000	0.0000000	-0.9372903
Se	-0.8950772	1.5790344	-2.3190480
Se	0.5290221	-1.7520910	2.0214685
Se	0.0000000	0.0000000	3.3801075
Se	-0.5290221	1.7520910	2.0214685
F	1.0722715	2.3813982	1.6454660
F	0.5080807	2.6196162	-2.5692951
F	-0.5080807	-2.6196162	-2.5692951
F	-1.0722715	-2.3813982	1.6454660

23.

C	-1.8213716	-1.8808277	-2.4246815
C	1.7887421	-1.3697070	1.7971842
C	-1.7887421	1.3697070	1.7971842
C	1.8213716	1.8808277	-2.4246815
N	-2.9815232	-1.9287510	-2.6210844
N	1.7487160	-2.4244253	1.2723763
N	-1.7487160	2.4244253	1.2723763
N	2.9815232	1.9287510	-2.6210844
Se	1.7689160	0.3199782	2.4978403
Se	0.0000000	-0.0000000	-0.7215732
Se	-0.0107238	-1.8296461	-2.1448751
Se	0.0000000	-0.0000000	3.9680537
Se	-1.7689160	-0.3199782	2.4978403
Se	0.0107238	1.8296461	-2.1448751

24.

C	0.4284137	2.6756025	-2.4017711
C	-0.4284137	-2.6756025	-2.4017711
C	-0.2284455	-2.8095434	2.1055620
C	0.2284455	2.8095434	2.1055620
F	-0.8754547	-3.2229623	-1.2717545
F	-1.4631108	-2.0672372	-2.9876588
F	-0.0107526	-3.6589888	-3.2080489
F	0.2532658	-3.7930138	1.3417834
F	-1.3545470	-2.3541950	1.5564166
F	-0.5392754	-3.3170163	3.2987657
F	1.3545470	2.3541950	1.5564166
F	-0.2532658	3.7930138	1.3417834
F	0.5392754	3.3170163	3.2987657
F	0.8754547	3.2229623	-1.2717545
F	1.4631108	2.0672372	-2.9876588
F	0.0107526	3.6589888	-3.2080489
Se	0.0000000	-0.0000000	3.6156473
Se	-1.1598895	1.4238668	2.2207497
Se	1.1598895	-1.4238668	2.2207497
Se	0.0000000	0.0000000	-0.6895431
Se	-1.0832992	1.4605114	-2.1170962
Se	1.0832992	-1.4605114	-2.1170962

25.

S	1.8570061	1.2237361	0.0683750
S	0.1246684	1.5733381	-0.9752187
S	-1.2047465	2.4070655	0.3467833
S	-1.8570061	-1.2237361	-0.0683750
S	-0.1246684	-1.5733381	0.9752187

S	1.2047465	-2.4070655	-0.3467833
H	-2.4794886	-2.3760205	0.2345457
H	0.8626453	-3.6944257	-0.1686528
H	-0.8626453	3.6944257	0.1686528
H	2.4794886	2.3760205	-0.2345457

26.

S	1.6585225	1.1026645	0.0564898
S	-0.0196086	1.5880496	-0.9617784
S	-1.2678991	2.5117074	0.3081301
S	-1.6585225	-1.1026645	-0.0564898
S	0.0196086	-1.5880496	0.9617784
S	1.2678991	-2.5117074	-0.3081301
F	2.6376329	2.3603352	-0.2640481
F	-0.8694106	4.0811760	0.1669635
F	-2.6376329	-2.3603352	0.2640481
F	0.8694106	-4.0811760	-0.1669635

27.

S	1.6237253	1.3751546	0.2989205
S	0.0573719	1.5506940	-1.0257523
S	-1.5004858	2.3164669	0.0767226
S	-1.6237253	-1.3751546	-0.2989205
S	-0.0573719	-1.5506940	1.0257523
S	1.5004858	-2.3164669	-0.0767226
C	1.1819736	-3.9751726	0.0335948
C	-2.4171604	-2.8472463	-0.0297434
C	2.4171604	2.8472463	0.0297434
C	-1.1819736	3.9751726	-0.0335948

N	-3.0125210	-3.8529214	0.1153574
N	0.9963271	-5.1375000	0.0715047
N	3.0125210	3.8529214	-0.1153574
N	-0.9963271	5.1375000	-0.0715047

28.

S	1.6289545	1.3859949	0.2636243
S	0.0112219	1.6023254	-0.9745320
S	-1.5620266	2.1766146	0.1835216
S	-1.6289545	-1.3859949	-0.2636243
S	-0.0112219	-1.6023254	0.9745320
S	1.5620266	-2.1766146	-0.1835216
C	-1.4692253	4.0013614	0.1371347
C	1.4692253	-4.0013614	-0.1371347
C	2.5735319	2.8795757	-0.1857925
C	-2.5735319	-2.8795757	0.1857925
F	1.1514580	-4.4736885	1.0638765
F	-1.1514580	4.4736885	-1.0638765
F	2.6855710	-4.4478077	-0.4696440
F	-2.6855710	4.4478077	0.4696440
F	0.5979314	-4.4918438	-1.0166549
F	-0.5979314	4.4918438	1.0166549
F	-2.8816641	-2.9139381	1.4830021
F	2.8816641	2.9139381	-1.4830021
F	-3.7094015	-2.8335193	-0.5174046
F	3.7094015	2.8335193	0.5174046
F	1.9384059	4.0107214	0.1055781
F	-1.9384059	-4.0107214	-0.1055781

29.

H	-2.6890469	-2.4164905	0.2823381
H	0.9721050	-3.8903589	-0.2106485
H	-0.9721050	3.8903589	0.2106485
H	2.6890469	2.4164905	-0.2823381
Se	-2.0034833	-1.1894984	-0.1406184
Se	-1.4033250	2.5013054	0.3986723
Se	0.0912016	1.5762682	-1.1051204
Se	-0.0912016	-1.5762682	1.1051204
Se	1.4033250	-2.5013054	-0.3986723
Se	2.0034833	1.1894984	0.1406184

30.

Se	1.8173175	0.7990696	0.1655869
Se	-0.0284299	1.4069874	-1.1180124
Se	-1.5026728	2.2981045	0.3637546
Se	-1.8173175	-0.7990696	-0.1655869
Se	0.0284299	-1.4069874	1.1180124
Se	1.5026728	-2.2981045	-0.3637546
F	2.9235404	2.0124433	-0.5087324
F	-1.0210408	4.0013541	0.3543508
F	-2.9235404	-2.0124433	0.5087324
F	1.0210408	-4.0013541	-0.3543508

31.

Se	1.9063109	1.0717956	0.4260577
Se	0.2063879	1.5873424	-1.0698869
Se	-1.4073565	2.5298532	0.2974919
Se	-1.9063109	-1.0717956	-0.4260577
Se	-0.2063879	-1.5873424	1.0698869

Se	1.4073565	-2.5298532	-0.2974919
C	0.7455750	-4.2386336	-0.3415862
C	-2.9927590	-2.5007457	-0.0371428
C	-0.7455750	4.2386336	0.3415862
C	2.9927590	2.5007457	0.0371428
N	0.3464188	-5.3444508	-0.4098412
N	-3.7336167	-3.3904640	0.1779451
N	-0.3464188	5.3444508	0.4098412
N	3.7336167	3.3904640	-0.1779451

32.

C	-1.5508435	4.1585858	0.1518005
C	1.5508435	-4.1585858	-0.1518005
C	2.7104000	2.9989736	-0.1829597
C	-2.7104000	-2.9989736	0.1829597
F	1.2885381	-4.6417973	1.0601527
F	-1.2885381	4.6417973	-1.0601527
F	2.6964046	-4.6981169	-0.5871057
F	-2.6964046	4.6981169	0.5871057
F	0.5740346	-4.5491319	-0.9688968
F	-0.5740346	4.5491319	0.9688968
F	-3.0624053	-2.9683018	1.4703512
F	3.0624053	2.9683018	-1.4703512
F	-3.8256603	-3.0518392	-0.5549967
F	3.8256603	3.0518392	0.5549967
F	2.0182706	4.1167564	0.0182738
F	-2.0182706	-4.1167564	-0.0182738
Se	-1.7400751	-1.3784603	-0.3658205
Se	-1.8009768	2.2073630	0.1609015

Se	-0.0055641	1.5870933	-1.1345281
Se	0.0055641	-1.5870933	1.1345281
Se	1.8009768	-2.2073630	-0.1609015
Se	1.7400751	1.3784603	0.3658205

CSD codes

Trisulphide compounds: UBADIN, VIXBEK, TIZHEQ, BIBPEH, COVHUQ, DAHDOF, DESYAB, HIHROFO1, RIKXIS, TCLMES.

Triselenide compounds: FUZGEO, KECFON, QUWDUI, SADYIF, VAWJOS, VAWJUY, XUNHUL, YADMOG.
